# Feature-level ensemble approach for COVID-19 detection using chest X-ray images

**DOI:** 10.1371/journal.pone.0268430

**Published:** 2022-07-14

**Authors:** Thi Kieu Khanh Ho, Jeonghwan Gwak

**Affiliations:** 1 Department of Software, Korea National University of Transportation, Chungju, South Korea; 2 Department of Biomedical Engineering, Korea National University of Transportation, Chungju, South Korea; 3 Department of AI Robotics Engineering, Korea National University of Transportation, Chungju, South Korea; 4 Department of IT & Energy Convergence (BK21 FOUR), Korea National University of Transportation, Chungju, South Korea; University of Engineering & Technology, Taxila, PAKISTAN

## Abstract

Severe acute respiratory syndrome coronavirus 2 (SARS CoV-2), also known as the coronavirus disease 2019 (COVID-19), has threatened many human beings around the world and capsized economies at unprecedented magnitudes. Therefore, the detection of this disease using chest X-ray modalities has played a pivotal role in producing fast and accurate medical diagnoses, especially in countries that are unable to afford laboratory testing kits. However, identifying and distinguishing COVID-19 from virtually similar thoracic abnormalities utilizing medical images is challenging because it is time-consuming, demanding, and susceptible to human-based errors. Therefore, artificial-intelligence-driven automated diagnoses, which excludes direct human intervention, may potentially be used to achieve consistently accurate performances. In this study, we aimed to (i) obtain a customized dataset composed of a relatively small number of images collected from publicly available datasets; (ii) present the efficient integration of the shallow handcrafted features obtained from local descriptors, radiomics features specialized for medical images, and deep features aggregated from pre-trained deep learning architectures; and (iii) distinguish COVID-19 patients from healthy controls and pneumonia patients using a collection of conventional machine learning classifiers. By conducting extensive experiments, we demonstrated that the feature-based ensemble approach provided the best classification metrics, and this approach explicitly outperformed schemes that used only either local, radiomic, or deep features. In addition, our proposed method achieved state-of-the-art multi-class classification results compared to the baseline reference for the currently available COVID-19 datasets.

## Introduction

The COVID-19 pandemic continues to cause detrimental consequences towards the global population, as evidenced by the ever-increasing number of deaths that are mainly caused by the lack of particular treatments and vaccinations for this disease. Although the fatality rate of the disease is approximately 2% to 3% [1], its rapid spread among humans, difficult identification during its inactive stage, and difficult differentiation from the common flu are of significant concern. At present, reverse transcription polymerase chain reaction (RT-PCR) tests [2] and antibody testing [3] are considered to be the most precise techniques to cope with COVID-19 challenges. However, while the former demands a sophisticated, costly, and time-consuming process to eliminate human-bias errors and health risks, the latter does not safeguard towards early detection and containment due to the uncertainty involved in the generation of antibodies before a week has elapsed from the initial infection. Therefore, researchers have been striving to produce fast, inexpensive, and reliable detection methods since the outbreak of this disease. Medical imaging presents a prominent solution owing to the ease with which abnormalities may be detected within the lungs, which are the first organs affected by COVID-19. Therefore, radiography images may possibly provide insight into lung conditions, leading to the subsequent identification of COVID-19. Two medical imaging techniques, namely X-ray and computerized tomography (CT)-scan modalities, are ubiquitously employed to diagnose COVID-19 [4, 5].

Despite possessing the advantages of being affordable and low-risk considering radiation hazards towards human health, X-ray modalities typically require radiologists for the identification of white spots containing water and pus, which may not result solely from COVID-19. Radiologists may mistakenly identify other thorax diseases, such as pulmonary tuberculosis, as COVID-19 [6]. Additionally, the similarity between COVID-19-affected, normal, and pneumonia-affected lung images increases the difficulty for radiologists to obtain a unanimous COVID-19 diagnosis. More importantly, manual interpretations significantly suffer from inter- and intra-radiologist variance, as well as influences from a variety of subjective factors, such as the level of experience, emotion, or fatigue of a radiologist. Conversely, CT images, which do not exhibit the high error rate obtained with the use of X-ray procedures, provide a more accurate detection method [7]. This modality is far more expensive than X-rays due to the requirement of large workloads from physicians and radiologists to correctly analyze a large volume of CT-scan images for each patient. Therefore, technological advances in artificial intelligence (AI), in particular deep learning (DL) approaches, indicate the promising use of computer-aided diagnosis (CAD) to overcome the aforementioned issues. These techniques may be used to learn high-dimensional features and achieve highly reliable performances compared to conventional methods of disease diagnosis.

In the current era of machine learning, DL techniques have achieved state-of-the-art performances in diverse tasks by reaching human-level accuracies [8], including medical image analysis [9]. In particular, DL techniques have successfully been used to diagnose various diseases including the detection of brain tumors from MRI images [10, 11], multiple types of brain disorders from electroencephalograms (EEGs) [12, 13], the levels of mental workloads using functional near-infrared spectroscopy (fNIRS) [14, 15], breast cancer from mammographic images [16, 17], and lung diseases from chest X-ray (CXR) images [18–23]. Accordingly, DL-based approaches using limited numbers of available CXR datasets for the COVID-19 classification task have been actively investigated [24–29]. However, there is a number of significant challenges that researchers have encountered when designing and implementing novel techniques to accurately diagnose and predict COVID-19.

First, the unavailability and quality of COVID-19 radiographic images strongly affects the detection model. Most previous studies used the available datasets that contained at most several hundreds of confirmed COVID-19 CXR images. As a result, poor predictions were generated due to over-fitting and increased generalization errors. It is therefore crucial to use data augmentation (DA) techniques for both the training and validation stages. Second, a common problem faced while working on COVID-19 tasks is class imbalance. While data from healthy control (HC) subjects and pneumonia patients are available on a large scale, COVID-19 remains as a minority class, which makes a model prone to providing unreliable prediction results. Therefore, re-sampling this dataset or generating a customized balanced dataset is necessary. Third, to enhance the classification accuracy and maximize the reliability of a model’s performance, preprocessing steps such as cropping, denoising, or histogram equalization to remove artifacts such as wires, probes, and augment the image contrast are required. Fourth, COVID-19 generally presents very similar symptoms to viral pneumonia, and it is difficult to differentiate between these diseases. Mild COVID-19 cases also show no indicators or specific symptoms that may be observed by the naked eye, leading to the classification of images obtained from such patients as normal images. Therefore, the use of three classes is vitally important to develop our proposed method. Finally, from the currently existing reports on radiographic images, the transferring of deep features extracted from pretrained networks is preferable, including those from ResNet, GoogleNet and AlexNet trained on the ImageNet database, which differs entirely from the properties of medical images. Considering these issues, the swift diagnosis and classification of COVID-19 from other thorax diseases using medical images remains challenging. Therefore, in this study, we aim to develop a robust method that is highly accurate for multi-class classification tasks using a limited dataset.

A significant number of studies in diverse image-level fields have demonstrated the promising capabilities of feature-level ensemble approaches [18, 30–33]. Moreover, local handcrafted features yield necessary gradient, orientation, and color- and pixel-based scale information. The use of only these features may rely heavily on local descriptors, which constrains the generalization ability of the resulting system. Meanwhile, radiomic features may be used to identify disease characteristics that are difficult to observe using the naked eye. Finally, deep features, as high dimensional level features, may be extracted to obtain substantial information from an original dataset. These findings motivate us to use all three types of features, which are deemed to be the predominant factors in CXR images for the classification of COVID-19.

In this study, we investigate the capability of the use of a feature-level ensemble approach for COVID-19 diagnosis. To achieve this, we develop a framework to obtain efficient and distinguishable features from local descriptors, radiomics algorithms, and deep learning models. Then, the potential of the feature-based ensemble approach is demonstrated using a multi-class classification task involving HC, pneumonia, and confirmed COVID-19 subject groups. The main contributions of this study are summarized as follows:

- Despite using a relatively small and balanced CXR dataset, we obtain a set of important features utilized for discriminating COVID-19 patients from HC and pneumonia patients.
- We thoroughly extract and evaluate distinguishable features, including shallow handcrafted features obtained from local descriptors, radiomics features specialized for radiographic medical images and collected from data-characterization algorithms, and deep features from pre-trained ResNet18 [34] and DenseNet121 [35]. The best optimal features are then concatenated to increase the classification accuracy of the developed system.
- We feed learned features into a collection of conventional machine learning classifiers and comprehensively compare the obtained classification results.

The remainder of the paper is organized as follows. In Section 2, we present related works on DL-based methods for COVID-19 classification tasks and discuss relevant challenges. Section 3 presents a description of our proposed framework for feature extraction, multiple feature integration, and multi-class classification problems. In Section 4, we introduce the publicly available CXR datasets that are used in this study and summarize the experimental results. Finally, we conclude the paper and provide future research directions in Section 5.

## Related works

As a significant branch of AI, DL has been proven to provide robust performances in terms of the classification, segmentation, and prediction of abnormalities in radiographic medical images. Specifically, various DL architectures have been extensively used to precisely diagnose COVID-19 utilizing diverse public datasets [36–38]. These architectures consist of convolutional neural networks (CNNs), autoencoders (AEs), recurrent neural networks (RNNs), generative adversarial networks (GANs), deep belief networks (DBNs), and hybrid networks such as CNN-AE and CNN-RNN for the automated detection of COVID-19. To accurately compare previous works with the present study, we present those works related to the detection of COVID-19, which is virtually modeled as a classification task of three classes: HC, pneumonia, and COVID-19 cases, which have been considered utilizing various DL approaches. Among them, transfer learning is considered the most common training scheme for COVID-19 detection. Knowledge is acquired from the training dataset on ImageNet [39] and then fine-tuned using a COVID-19 classification task with a CXR dataset, thereby yielding a faster convergence and improved performance. Currently existing pretrained deep-CNN networks such as VGGNet, ResNet, and DenseNet are popular for COVID-19 detection [26, 28, 40–45].

By combing predictions obtained from multiple models to reduce generalization errors and variance, as well as to generate more accurate detection results, ensemble learning has been adopted for COVID-19 classification problems using CXR images. Goodwin et al. [46] integrated predictions from 12 models (Resnet18,50,101,152; WideResnet50,101; ResNeXt50,101; MobileNet-v1; Densenet121,169,201) to achieve more accurate results. In addition, Karim et al. [47] ensembled three models (ResNet18, VGG19, and DenseNet161) to demonstrate the improved performance of this approach compared to the results obtained by simply training only a single model. Misra et al. [42] also investigated this technique by first using only one model such as ResNet18, followed by further fine-tuning of this model using three different datasets, and finally combining three networks to obtain superior classification results.

Because CXR COVID-19 images have distinguished distributions while still presenting similar characteristics to CXR images of pneumonia patients, the domain adaptation technique can be applied. Zhang et al. [48] created a COVID-DA in which the discrepancy between the data distribution and task was handled by utilizing feature adversarial adaptation and a classifier scheme. They showed that this learning framework noticeably provided better COVID-19 detection results. Moreover, to overcome the challenge of data scarcity, cascaded network architectures have been introduced to lessen the occurrence of overfitting. LV et al. [27] classified COVID-19 samples by cascading two networks (ResNet50 and DenseNet169). In particular, once an image was classified as viral pneumonia from HC, bacterial pneumonia, and viral pneumonia subjects using ResNet, it was then fed into DenseNet169 for the COVID-19 classification task. The infectious regions were concentrated based on an attentional mechanism referred to as Squeeze-Excitation. The image quality and its features were ameliorated using a contrast limited adaptive histogram equalization technique and an additional module excitation, respectively. Their obtained results from both cascaded networks achieved significantly high accuracies.

Wang et al. [49] designed a COVID-Net architecture and fabricated a customized dataset for training purposes. They obtained a reliable COVID-19 detection accuracy of 93.3% by incorporating a lightweight design pattern, selective long-range connectivity, and architectural diversity. Their approach of optimizing the COVIDx dataset using DA techniques and pretrained deep models also highly contributed to increase the accuracy of the resulting system. Moreover, while Ozturk et al. [50] designed Dark-COVIDNet based on the foundation of DarkNet-19 to utilize convolutional layers with different filters on each layer, Punn et al. [44] proposed NASNetLarge for automated COVID-19 detection with comparable results. Additionally, Oh et al. [29] addressed the problem of a limited amount of data by proposing the statistical analysis of potential imaging biomarkers, in addition to a patch-based CNN to achieve high accuracies. They also provided clinically interpretable saliency maps with the hope that they would prove useful for further COVID-19 diagnosis techniques and patient triage.

Inspired by these successful systems, while also noting their most common obstacles, we aimed to develop a novel model for COVID-19 multi-class classification. Because feature-based concatenation that is achieved using three feature types, including handcrafted, radiomic, and deep features, has not yet been thoroughly evaluated for COVID-19 classification tasks, we proposed an extensive framework to address the challenging diagnosis of COVID-19 using CXR images.

## Materials and methods

### Feature extraction

**Fig 1:**
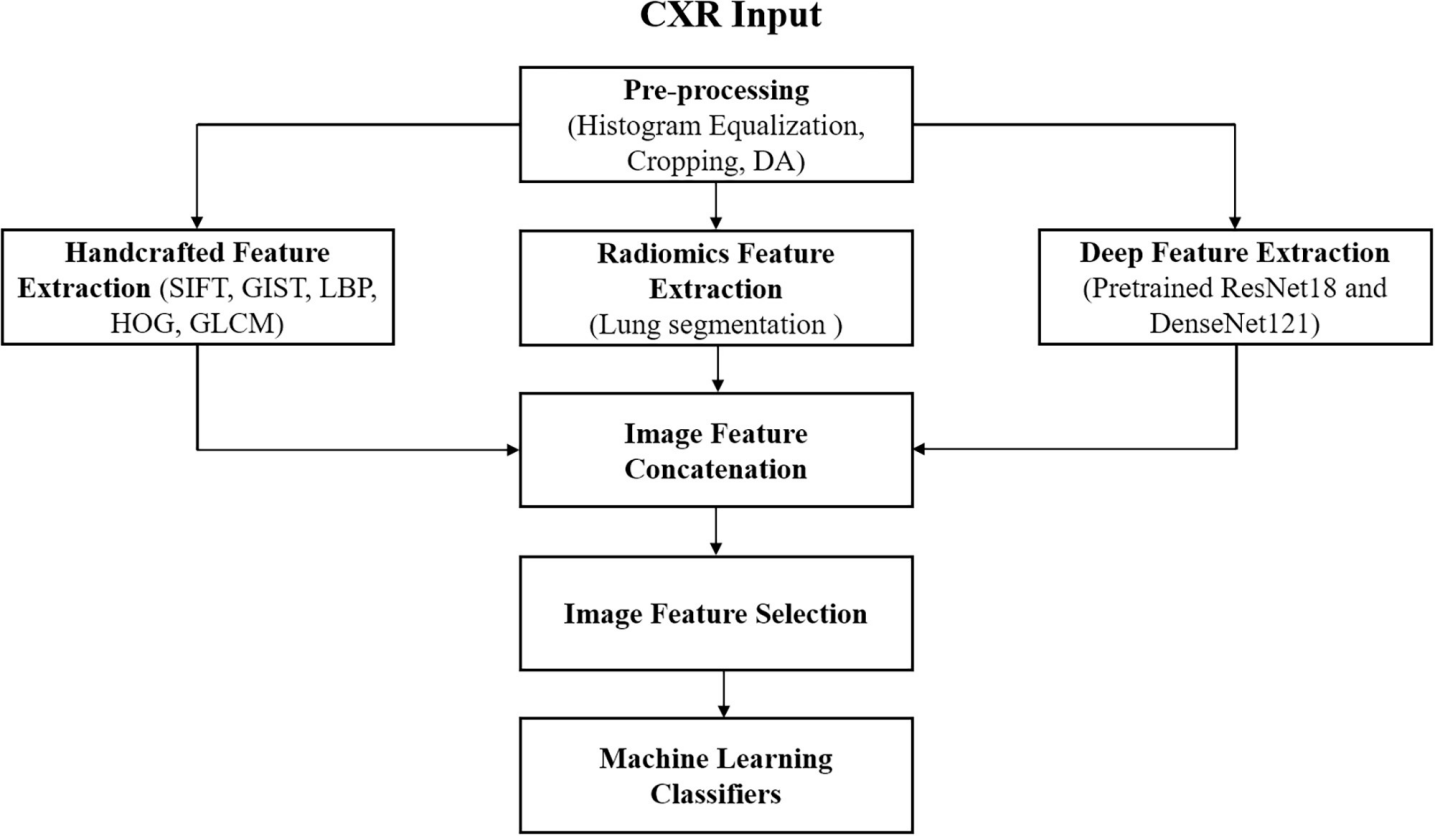
Overview of the proposed method for our COVID-19 study.

Fig 1 presents an overview of our proposed approach. We first preprocessed the input image using preliminary steps, including histogram equalization, cropping, horizontal flipping, and batch augmentation methods. Second, we manually extracted handcrafted features using five types of local descriptors, radiomic features using data-characterization feature extractors with the essential step of lung segmentation, and deep features using pretrained ResNet18 on ImageNet and pretrained DenseNet121 on a 14-thoracic-disease-classification task from our previous works [18]. Lastly, we concatenated all features and selected the best optimal features, which were further fed into conventional machine learning classifiers to obtain the highest classification metrics. All feature extraction steps were implemented using MATLAB 2020a.

#### Handcrafted feature extraction

In this stage, we utilized five distinguished types of local feature descriptors to effectively learn medical image information from different facets. The scale-invariant feature transform (SIFT) [51] extracts structural information over the entire range of the scale and exhibits rotation-invariant features. Four main steps are involved, including scale-space peal selection, key point localization, orientation assignment, and key point descriptor creation and matching. Therefore, this system is robust towards the extraction of individual local features, although the histogram of gradients computation requires significant time. GIST [52] summarizes the gradient information, including the scales and orientations from different parts of an image. GIST features can represent the dominant spatial structure of an image using a set of perceptual dimensions that contain substantial information for identifying the scene in a CXR image. The local binary patterns (LBP) feature descriptor [53] computes a local representation of the texture of an image by comparing each pixel with its pixel neighbors and considers the result as a binary number. The LBP texture operator is efficient due to its discriminative power and computational simplicity. The histogram of oriented gradients (HOG) feature descriptor [54], unlike the local image SIFT descriptor, is a regional receptive field histogram defined over subregions in the image domain. It is essentially computed on a dense grid of uniformly spaced cells, and it then uses the overlapping of the local contrast normalization to improve its feature extraction performance. The gray level co-occurrence matrix (GLCM) [55] is a statistical method that examines the texture characteristics of an image based on the spatial relationships of the pixels. Given a medical image composed of pixels with a specific gray level, it calculates how often pairs of pixels within a specific range of values in the image appear that exhibit a particular spatial relationship to generate a GLCM matrix, and it thus extracts statistical measures for this matrix.

#### Radiomic feature extraction

Given medical images, radiomics methods may be used to convert them into high-dimensional, mineable, and quantitative features using data-characterization extraction algorithms, which may be used to enhance the decision-support of radiographic studies. Studies on handcrafted radiomics features are generally composed of five main steps: (1) preprocessing for the reduction of noise and artifacts coupled with image smoothing and enhancement methods; (2) segmentation, which is a critically important step for highlighting the features from the pixels of tumorous regions; (3) intensity and gradient feature extraction; (4) feature reduction for eliminating redundant features that are highly correlated and irrelevant to the task, which contribute towards the over-fitting of the designed models; and (5) statistical analysis for feature evaluation and use in a particular application. Previous works showed that CT has been the most widely used imaging modality in radiomics research, which is used to quantify the tissue density [56]. This method is followed by magnetic resonance imaging (MRI), which provides better image contrast, apparent multiplanar capacity, and fewer radiation artifacts, allowing for the detection of the density of a tumor and determination of the tumor characteristics [57]. To the best of our knowledge, existing studies [18] only extracted either local features or deep features, but not a combination of the above two features coupled with radiomic features in the CXR images, such as considering current COVID-19 research.

**Fig 2:**
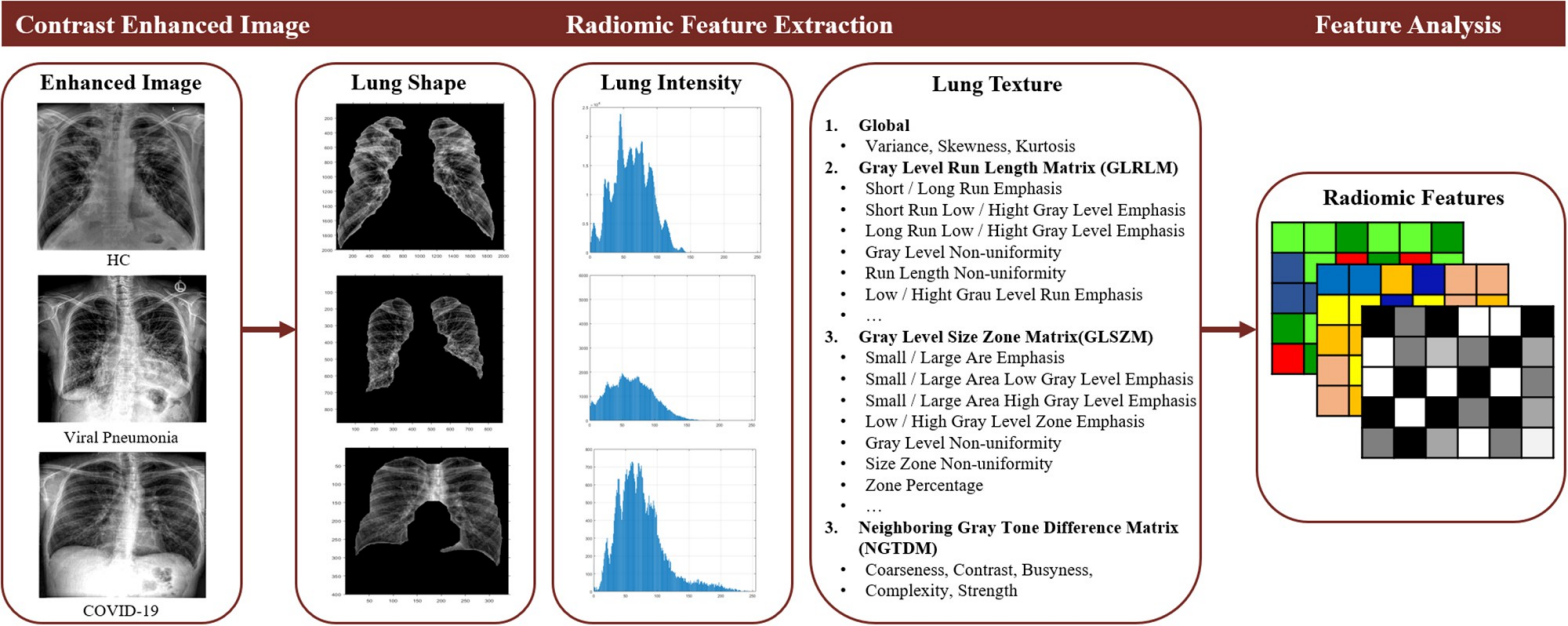
Flowchart of radiomic feature extraction.

To describe the unique prognostic and diagnostic features in our CXR dataset, it is essential to include both feature extraction to precisely describe the collected data with as many features as possible and feature selection to generalize distinctive patterns within the data by eliminating redundant information at this stage. Therefore, a total of 46 features were extracted from the lung regions of each image, including 12 statistical non-texture features; 3 normalized intensity features; 13 gray level run length matrix (GLRLM) features, which quantify the gray level run defined by the length of the consecutive pixels at the same gray level value; 13 gray level size zone matrix (GLSZM) features, which define the number of connected voxels that share the same gray level intensity; and 5 neighboring gray-tone difference matrix (NGTDM) features, which measure the difference between a gray value and the average gray valued computed using its neighbors. Fig 2 illustrates a flowchart of the radiomic feature extraction method.

#### Deep feature extraction

Because the input image goes through multiple convolution layers, which allows us to obtain high-level features, and each layer receives additional inputs from all preceding layers and passes on its own feature maps to all subsequent layers, we used two types of pre-trained deep models for the deep feature extraction stage. First, we adopted the relatively lightweight ResNet18 trained on ImageNet to avoid the overfitting problem. This is achievable because an overly complex model is typically susceptible to overfitting when it is trained using a small dataset, such as our customized COVID-19 dataset. Second, to appreciably boost the classification accuracy, we re-used the pretrained DenseNet121 model obtained from our former 14-lung class classification task owing to the advantage of this model, which has learned CXR features. Compared to other pretrained models, each layer of DenseNet121 obtains a cumulative knowledge of information and gradients from previous layers. The features are then passed to subsequent layers and finally concatenated into the depth dimension. The resulting network can therefore be thin and compact with few layers, while it can also learn diversified X-ray feature patterns. We found that this transfer learning scheme for both pretrained deep models was quite stable during the training process.

### Conventional machine learning classifiers

Machine learning (ML) techniques have been successfully utilized in medical applications [58]. It is arduous to conclude which ML classifier is superior to others from among various available ML algorithms since their suitability and performance depend on the application and the nature of the dataset. For instance, simpler ML algorithms with high bias and low variance learn better from small datasets and avoid overfitting. Thus, to quantitatively check the efficiency of our feature extraction methods, we chose a set of relatively simple ML classifiers to accomplish the three-class classification task using the obtained integrated features. Chosen ML classifiers are described below.

- Linear Discriminant Analysis (LDA) [59]: Given a set of samples belonging to three CXR classes, we determined the intra- and inter-class to obtain a linear transformation by solving the generalized eigenvalues. The Euclidean distance was optimized after 200 epochs with discriminant features to perform the classification task on the transformed space.
- k-Nearest Neighbors (kNN) [60]: This algorithm ran several times with an initial value of *K* = 3 to determine the distance between each data point and cluster centroid using the Euclidean distance. Not only did kNN not require tuning parameters and the prior supposition of the data, it was also able to determine the distance required for the classification task after 200 epochs.
- Gaussian Naive Bayes (GNB) [61]: As a probabilistic approach, this classifier computed the probability for each class using the Bayesian rule. In addition, it stored the means and standard deviations of the input variables for each class. GNB was the easiest and simplest classifier among the eight types.
- Support Vector Machine (SVM) [62]: A sigmoid kernel *k*(*x*, *y*) = *tanh*(*αx*^*T*^ *y* + *x*) was used that allowed SVM to construct decision hyperplanes to separate three feature classes. This kernel imitates the concept of 2-layer perception, and it generated fewer training errors on non-linear features in comparison with other kernels (linear, polynomial, and Gaussian).
- Adaptive Boosting (AdaBoost) [63]: A set of 1000 weak classifiers was initially trained to determine the misclassified data points, and the weights of these points were increased using an iterative procedure. We set the number of estimators to *n* = 200, and this process possibly obtained a higher precision compared to the other classifiers.
- Random Forest (RF) [64]: We set the tree’s max depth as 8, the number of estimators as 200, and ran RF several times because it was constructed using an ensemble of decision trees. Each sample was voted on by each decision tree and the sample with the most votes was provided as the final prediction. Using this method, it was easy to observe the deterministic behavior of this classifier during the fitting of the RF algorithm.
- Ensemble Learning (Ensemble) [65]: We combined the above six-base learners to obtain a strong integration by selecting the feature outputs of the base-level model as a meta-classifier. The final class was determined by the hard-voting approach. The classification accuracy was undoubtedly either equal to or higher than the best accuracy of the base learners.
- XGBoost [66]: Unlike RF, XGBoost is known as a decision-tree-based ensemble algorithm that uses a gradient boosting framework. This ensemble model created a strong classifier based on previous weak learners by using an iterative procedure. The errors of the previous predictors were corrected by the following model until the full set of training data was accurately predicted by the final optimal model. We set the tree’s max depth to 50, number of estimators to 80, and learning rate to 0.1
- Neural Network (NN) [67]: We manually ran NN with different parameter settings to obtain the best fit of the algorithm with our extracted features. The simple NN model included the input layer, three hidden layers, and the output layer in conjunction with an ReLU activation function, Adam optimizer, 1e-5 learning rate, and 500-iteration duration. We expected to achieve a higher classification accuracy than obtained using the other methods owing to the better generalization ability of this method.

## Experimental results

### CXR dataset and preprocessing steps

Given five public CXR-based COVID-19 datasets, including the COVID-19 Image Data Collection [68], Actualmed COVID-19 Chest X-ray Dataset Initiative [69], Fig 1 COVID-19 Chest X-ray Dataset Initiative [70], COVID-19 Radiography Database [71], and Extensive COVID-19 X-Ray and CT Chest Images Dataset [72], we selected the most clear and visible CXR images to obtain the best performance of the developed system. The dataset was collected based on two criteria. (i) It should be balanced, and include three classes (normal, pneumonia and COVID cases in which the pneumonia and COVID are quite similar at the context patterns on the chest X-ray images and their clinical symptoms—this would challenge any ML algorithms). (ii) Each patient should consist of only one X-ray image—as the reason why we tried to gather all five available datasets.

**Fig 3:**
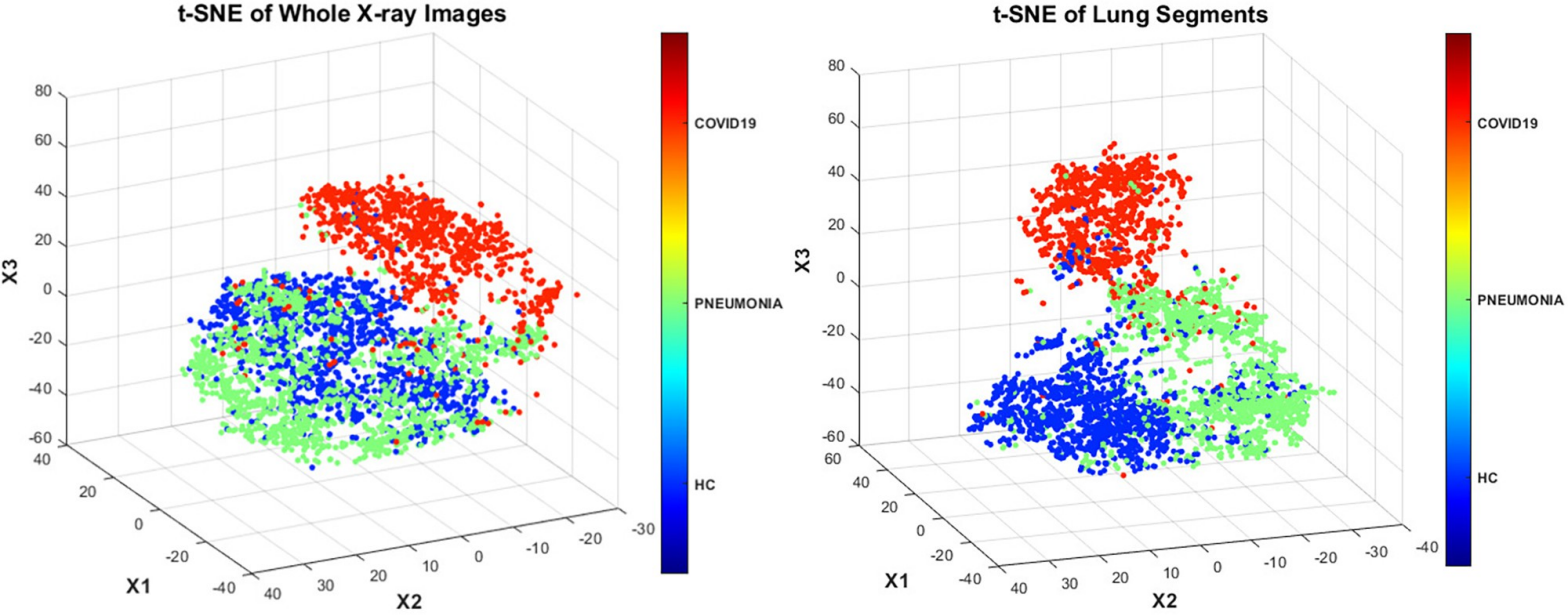
t-SNE dimensions of the class distributions using whole CXR images (left) and lung segments (right).

Table 1 summarizes our collected dataset, which was then randomly split by two approaches: (1) for handcrafted-, radiomic- and deep features to be fed to conventional ML classifiers, we split train/test (80/20) where 10-fold CV was applied in the training phase and remaining unseen 20% images would be tested after we obtained the best hyperparameters of ML classifiers by CV-based tuning hyperparameters. (2) for pretrained deep models, depending on the nature of the features and the model, we split the train/valid/test (70/10/20), where 10% of validation set was used to observe the training behavior and allowed us to stop the training once it was likely to overfitting, then it would generalize well on the unseen 20% test data. After spliting the data, to exclude any potential bias of the dataset, we employed several standardized preprocessing steps including cropping, histogram normalization, constant threshold contouring, and data augmentation techniques. We used the t-distributed stochastic neighboring entities (t-SNE) technique [73] to visualize the three-class distributions. When using handcrafted and deep features extracted from whole CXR images, it was relatively difficult to distinguish the HC subjects from pneumonia patients (Fig 3—left). Meanwhile, the lung segmentation step that was necessary for the radiomic feature extraction stage significantly assisted the classifiers such that they could easily discriminate all three classes (Fig 3—right).

**Table 1:** CXR data information.

**No. of Images:** Healthy Control (HC): 1341 images; Viral Pneumonia: 1345 images; COVID-19: 1093 images				
Approach	Training	Validation	Testing	Preprocessed Steps
Handcrafted, Radiomic and Deep Feature Integration	3028 (80%)	–	755 (20%)	Cropping, Histogram Equalization, Constant Threshold Contouring
Pre-trained ResNet18 and DenseNet121	2646 (70%)	378(10%)	764 (20%)	Cropping, histogram equalization, horizontal flipping, and batch augmentation methods in both training and validation

### Classification results

As previously mentioned, in the first approach, five types of handcrafted features obtained by SIFT, GIST, LBP, HOG, and GLCM descriptors; radiomic features aggregated by data-characterization algorithms; and deep features obtained from the pooling layer 5 (Pool5) of pretrained ResNet18 and from the convolutional layer 5 (Conv5) of pretrained DenseNet121 were selected to describe the image patches from different perspectives. The CXR data was accordingly split into training (80%) and testing (20%) subsets for the latter part of classification using traditional classifiers. In the second approach, the dataset was divided into training (70%), validation (10%), and testing (20%) subsets to fulfill three-class classification by directly using the ResNet18 system trained on ImageNet and the DenseNet121 system trained on the 14-lung abnormality classification task.

Table 2 summarizes the best classification metrics for each feature-level approachIn general, the feature selection approach outperformed all other approaches to achieve the highest accuracy of 0.941 via NN. Therefore, the classification results must be improved by carefully choosing appropriate and useful features, as demonstrated by the results of the present COVID-19 study. Tables 3 to 8 show each combination of features to provide a broad observation of our obtained results.

**Table 2:** Best classification metrics for each feature-level approach.

Approach	Handcrafted and Radiomic Features	Pretrained Resnet18	Pretrained DenseNet121	Combined Deep Features	All Features	Selected Handcrafted, Radiomic, and Deep Features
**Accuracy**	0.892	0.886	0.917	0.912	0.925	**0.941**
**Precision**	0.895	0.899	0.932	0.926	0.926	**0.944**
**Recall**	0.892	0.886	0.917	0.919	0.925	**0.941**
**F1 Score**	0.892	0.882	0.914	0.910	0.922	**0.940**

**Table 3:** Classification accuracy obtained using handcrafted and radiomic features.

Features	LDA	kNN	GNB	SVM	AdaBoost	RF	Ensemble	XGBoost	NN
SIFT	0.656	0.720	0.608	0.615	0.665	0.725	0.739	0.720	**0.780**
GIST	0.674	0.730	0.688	0.605	0.690	0.705	0.756	0.730	**0.764**
LBP	0.689	0.658	0.660	0.626	0.690	0.710	0.748	0.716	**0.791**
HOG	0.686	0.658	0.660	0.626	0.690	0.710	0.746	0.711	**0.790**
GLCM	0.699	0.722	0.679	0.657	0.724	0.751	0.769	0.738	**0.820**
Radiomics	0.769	0.838	0.745	0.727	0.764	0.830	0.849	0.841	**0.876**

**Table 4:** Classification accuracy obtained using all handcrafted and radiomic feature combinations.

Metrics	LDA	kNN	GNB	SVM	AdaBoost	RF	Ensemble	XGBoost	NN
Accuracy	0.792	0.832	0.746	0.773	0.765	0.853	0.847	0.844	**0.892**
Precision	0.809	0.835	0.849	0.725	0.786	0.828	0.856	0.861	**0.895**
Recall	0.792	0.832	0.746	0.773	0.765	0.853	0.847	0.844	**0.892**
F1 Score	0.774	0.832	0.737	0.735	0.750	0.817	0.829	0.823	**0.892**

**Table 5:** Classification accuracy obtained using selected handcrafted (LBP + HOG + GLCM) and radiomic feature combinations.

Metrics	LDA	kNN	GNB	SVM	AdaBoost	RF	Ensemble	XGBoost	NN
Accuracy	0.814	0.836	0.775	0.793	0.782	0.864	0.857	0.855	**0.882**
Precision	0.835	0.866	0.821	0.819	0.829	0.893	0.891	0.879	**0.893**
Recall	0.814	0.836	0.775	0.793	0.782	0.864	0.857	0.865	**0.882**
F1 Score	0.816	0.815	0.777	0.779	0.768	0.855	0.890	0.854	**0.888**

**Table 6:** Classification accuracy obtained using combined deep features (Pool5 of Resnet18 + Conv5 of Densenet121).

Metrics	LDA	kNN	GNB	SVM	AdaBoost	RF	Ensemble	XGBoost	NN
Accuracy	0.837	0.862	0.810	0.826	0.848	0.865	0.888	0.858	**0.912**
Precision	0.852	0.865	0.940	0.892	0.865	0.876	0.891	0.875	**0.926**
Recall	0.837	0.862	0.810	0.826	0.848	0.865	0.888	0.858	**0.919**
F1 Score	0.834	0.862	0.811	0.833	0.837	0.865	0.889	0.848	**0.910**

**Table 7:** Classification accuracy obtained using all features.

Metrics	LDA	kNN	GNB	SVM	AdaBoost	RF	Ensemble	XGBoost	NN
Accuracy	0.849	0.877	0.812	0.842	0.857	0.879	0.900	0.875	**0.925**
Precision	0.865	0.880	0.938	0.901	0.890	0.895	0.913	0.889	**0.926**
Recall	0.849	0.877	0.812	0.842	0.857	0.879	0.900	0.875	**0.925**
F1 Score	0.846	0.877	0.810	0.849	0.853	0.871	0.896	0.866	**0.922**

**Table 8:** Classification accuracy obtained using selected handcrafted, radiomic and deep features.

Metrics	LDA	kNN	GNB	SVM	AdaBoost	RF	Ensemble	XGBoost	NN
Accuracy	0.866	0.882	0.838	0.845	0.875	0.905	0.919	0.905	**0.941**
Precision	0.868	0.882	0.891	0.826	0.889	0.895	0.917	0.933	**0.944**
Recall	0.866	0.882	0.838	0.845	0.875	0.905	0.919	0.905	**0.941**
F1 Score	0.850	0.882	0.830	0.820	0.866	0.880	0.908	0.899	**0.940**

Table 3 summarizes the handcrafted and radiomic feature-based classification accuracies obtained using nine ML classifiers. As expected, considering the efficiencies of the different features and because the radiomics features consider a more significant number of radiographic features, almost all classification accuracies of these features are higher than those obtained using other handcrafted features. Moreover, it should be noted that the radiomics features utilized feature reduction to discard redundant information, which also improved the accuracies of these features. Considering the performances of the various classifiers, in average, LDA using discriminant features, SVM using hyperplanes, and GNB and AdaBoost using the Bayesian theorem were unable to easily separate the three classes on average. Conversely, kNN using a highly convoluted decision boundary to determine the distance metric and the growth of RF trees from bootstrap samples performed better in comparison to the aforementioned classifiers. Considering the ensemble learning method, which utilized a combination of heterogeneous weak learners to vote for the predicted classes, this method outperformed all six aforementioned classifiers. The ensemble classifier was less likely to overfit and produced a better generalizability, resulting in the second-highest accuracy of 0.849, followed by kNN, RF, LDA, AdaBoost, GNB, and SVM with accuracies of 0.838, 0.830, 0.769, 0.764, 0.745, and 0.727 using radiomic features, respectively.

Considering the other evaluated metrics, XGBoost, which is based on a gradient boosting scheme, was adequately robust to assist in fine-tuning and parameter regularization. From our observations, although XGBoost and RF seemingly resembled the decision-tree ensemble concept, these systems significantly differed. While RF constructed each tree independently and the results were combined at the end of the process using the majority rules, XGBoost built trees in a forward stage-wise manner and combined the obtained results during this process. Therefore, although XGBoost outperformed RF in the initial epochs and exhibited a more stable training curve, the final classification results of these two systems are relatively comparable. Finally, NN outperformed all other classifiers to achieve the highest accuracy obtained by radiomics features of 0.876, which indicated that NN was relatively flexible towards adopting sufficient feature patterns without requiring any feature-engineering steps or structured data, as are required by most conventional algorithms. Therefore, NN could automatically learn the high-level features in an end-to-end manner.

To evaluate the efficacy of the feature-level approach, we first either integrated all handcrafted and radiomic features, as shown in Table 4, or performed the feature selection step by selecting the handcrafted (LBP + HOG + GLCM) and radiomic features shown in Table 5. As shown by these tables and considering the characteristics of the selected discriminant features, we achieved better classification metrics compared to those obtained using the combination of all features, which indicates that feature selection is crucial for enhancing the performance of traditional classifiers. Additionally, the deep features, which were extracted by Pool5-ResNet18 and Conv5-DenseNet121, were combined. Table 6 shows the classification metrics obtained via this combination. Compared to handcrafted and radiomic feature ensemble, all deep feature-based classifiers demonstrated slightly higher classification performances. Moreover, to quantitively assess the different feature-level ensemble techniques, we combined all features and integrated the selected handcrafted, radiomic, and deep features, as demonstrated in Tables 7 and 8, respectively.

To efficiently compare our proposed approach with the reference COVID-19 baseline, we selected seven studies working on the three-class (HC, pneumonia, and COVID-19) classification task. Table 9 shows the dataset, deep learning architecture, preprocessing steps, and classification results of each study. For the classification tasks using Chest X-ray images, deep learning-based models are commonly required a bunch of data (i.e. public ChestX-ray14 dataset with more than 100,000 images). However, regarding the current COVID situation, the public dataset is very limited. For example, PDCOVIDNet [74] was introduced to detect COVID-19 from chest X-ray images by a dilated CNN. Their dataset included only 219 COVID-19 positive, but 1341 normal and 1345 viral pneumonia images. ECOVNet [75], known as an ensemble of CNN based on EfficientNet, was later proposed to detect COVID-19 from X-ray images. A total of 589 COVID-19 in a total of 13,914 chest X-ray images was used. Thus, data augmentation techniques were strongly required for these studies to surmount the challenges of imbalanced datasets as CNN-based architectures require sufficient amount of data for effective training; and thus they could outperform existing COVID-19 detection studies. However, in reality, non-resampled data (without data augmentation) is preferable to detect COVID-19 accurately (i.e., in real-time applications).

**Table 9:** Comparison of the three-class classification studies from previous CXR-based COVID-19 studies.

Work	Number of Cases	Preprocessing	Approach	Performance (%)
Wang et al. [49]	266 COVID-19 8066 HC 5538 Pneumonia	DA	COVID-Net	Accuracy = 93.3 Sensitivity = 91 PPV = 98.9
Ucar et al. [43]	76 COVID-19 1538 HC 4290 Pneumonia	DA RGB format Normalizing	COVIDiagnosis-Net	Accuracy = 98.3 Specificity = 99.13 F1-Score = 98.3
Ozturk et al. [50]	127 COVID-19 500 HC 500 Pneumonia	N/A	DarkCovidNet (CNN)	Accuracy = 87.02 Specificity = 92.18 Sensitivity = 95.35 Precision = 89.96 F1-Score = 87.37
Li et al. [21]	179 COVID-19 179 HC 179 Pneumonia	Create a Noisy Snapshot Dataset	KTD framework (DenseNet121, ShuffleNetV2, MobileNetV2)	Accuracy = 84.3 AUCROC = 94
Punn et al. [44]	108 COVID-19 453 HC 515 Pneumonia	Class Balancing Methods Binary Thresholding Adaptive Total Variation Method	NASNetLarge	Accuracy = 98 Specificity = 95 Precision = 88 F1-Score = 89
Elasnaoui et al. [22]	6087 images (2780 Bacterial Pneumonia, 1724 Coronavirus (1493 Viral Pneumonia, 231 COVID-19)) 1583 HC	Intensity Normalization CLAHE Method DA Resizing	Inception ResNetV2	Accuracy = 92.18 Specificity = 96.06 Sensitivity = 92.11 Precision = 92.38 F1-Score = 92.07
Khobahi et al. [23]	99 COVID-19 8851 HC 9579 Pneumonia	DA	CoroNet (TFEN + CIN modules)	Accuracy = 93.50 Sensitivity = 90 Precision = 93.63 F1-Score = 93.51
Chowdhury et al. [74]	219 COVID-19 1341 HC 1345 Pneumonia	DA	PDCOVIDNet (CNN)	Accuracy = 96.54 Precision = 96.58 Recall = 96.59 F1-Score = 96.58
Chowdhury et al. [75]	589 COVID-19 8851 HC 6053 Pneumonia	DA	ECOVNet (pre-trained EfficientNet)	Accuracy = 94.68 Precision = 94.76 Recall = 94.68 F1-Score = 94.70
Perumal et al. [76]	183 COVID-19 8066 HC 5538 Pneumonia	N/A	INASNET (Inception Nasnet)	Accuracy = 94.3 Precision = 94.0 Recall = 94.0 F1-Score = 94.0
**Proposed method**	1093 COVID-19 1341 HC 1345 Pneumonia	Cropping, DA Histogram Equalization Constant Threshold Contouring	Feature-based Ensemble	Accuracy = 94.1 Precision = 94.5 Recall = 94.1 F1-Score = 94.0

As a result, we attempted to build a framework to deal with small datasets. However, our dataset (espically COVID-19 class) is mostly bigger than others mentioned in the Table 9 since the dataset should be balanced, and include three classes (normal, pneumonia and COVID cases in which the pneumonia and COVID are quite similar at the context patterns on the chest X-ray images and their clinical symptoms—this would challenge any ML algorithms). It is shown that our method achieved consistent classification performance and outperformed most of other methods due to our customized and balanced dataset, with the exception of those methods using extremely imbalanced datasets. This may indicate that some issues existed in these previous studies. For example, an imbalance of the class distribution typically causes issues for most classifiers. A model may predict that all samples belong to majority classes (i.e., HC or pneumonia) due to its poor generalization ability, or it may be prone to overfitting during training. Despite the use of DA techniques to increase the number of COVID-19 samples, classification metrics (such as the sensitivity, specificity, precision, recall, F1-score, and AUC) would lose their meanings when measuring the prediction ability of a model, resulting in the so-called “cost-sensitivity of misclassification errors” problem.

## Conclusions and future works

Over a short period of time, the emerging coronavirus COVID-19 pandemic has significantly endangered the health of many people throughout the world. Fortunately, the continuous and conscientious efforts of researchers to develop new methods, such as those involving deep learning techniques, that utilize CXR data have resulted in significant progress for the effective detection of COVID-19. However, the current collection of large datasets for training deep learning networks are burdensome due to the absence of a benchmark dataset. In this study, we introduced a feature-level integration approach for a multi-class classification task using a relatively small customized CXR dataset. We thoroughly investigated different feature patterns that were extracted by local descriptors, radiomics algorithms, and pre-trained deep models to achieve deeper insights into the critical factors that affect the differentiation between COVID-19 cases, HC, and pneumonia patients. We also demonstrated that feature selection and combination are crucial factors that affect the ability of a system to accurately describe dataset information and ameliorate the classification results. In addition, our proposed method provided a competitive performance compared to the COVID-19 detection baseline.

Although we designed a comprehensive experiment in which the training dataset was assembled using various dataset resources to reduce bias and overfitting issues, compiling a dataset of adequate COVID-19 images from reliable and authentic sources was challenging. Therefore, the number of images used in this study was relatively small such that a class balance was achieved. Different preprocessing steps were required when utilizing this dataset, which may result in the overestimation of the performance of the developed model. Moreover, due to the unavailability of properly annotated data, we simply segmented the lung regions based on our knowledge without the confirmation of experienced radiologists for conducting the radiomics feature extraction process. Finally, the manual extraction of features from different perspectives (including handcrafted, radiomics, and deep features), performing feature selection and concatenation, and inputting the features into various ML classifiers was time consuming and resulted in significant computational costs.

Deep-learning-based radiomics (DLR) [77], which may also be referred to as “discovery radiomics”, is the process of extracting deep features from various deep architectures. An explicit advantage of DLR over our proposed radiomics framework is that the former does not require any prior knowledge, thereby allowing high-level features to be extracted in a completely automated fashion. Because deep learning networks can be trained in an end-to-end manner, their performance can be systematically improved as more training samples are supplied. Moreover, DLR significantly reduces computational time and costs by disregarding the segmentation step, which requires the creation of radiologist-based manual object annotations as other automatic segmentation methods are highly error-prone and result in inaccurate performances. More importantly, the diverse features extracted from the original and segmented images using the handcrafted intensity and gradient features are concatenated as the input of a deep network, and they are required to improve the performance of the resulting system. Therefore, DLR is a promising approach that will be investigated in our future studies. In addition, considering the promising results obtained by our proposed approach and the promising capabilities of the DLR method, we hope to improve the detection accuracy of COVID-19 by collecting a larger dataset than used in the present study. To aid clinicians to achieve the accurate screening of patients, as well as to ensure the reliability of deep learning techniques for COVID-19 diagnosis, obtaining COVID-19 ground-truth annotations and developing an accurate segmentation model and the related attention maps should also be addressed in future studies.
